# Femoral artery calcification predicts hip fracture in maintenance hemodialysis patients

**DOI:** 10.1007/s11657-025-01536-1

**Published:** 2025-08-09

**Authors:** Shun-Neng Hsu, Jhao-Jhuang Ding, Ping-Huang Tsai, Chia-Lin Yang, Chun-Liang Hsu, Yu-Juei Hsu

**Affiliations:** 1 Division of Nephrology, Department of Internal Medicine, Tri-Service General Hospital, National Defense Medical University, No 325, Section 2, Cheng-Kung Road, Neihu 114, Taipei, Taiwan; 2 Department of Pediatrics, Tri-Service General Hospital, National Defense Medical University, Taipei, Taiwan; 3 Orthopaedic Department, Tri-Service General Hospital, National Defense Medical University, Taipei, Taiwan; 4 Department of Biochemistry, National Defense Medical University, Taipei, Taiwan

**Keywords:** Femoral artery calcification, Hip fracture, Vascular calcification, Dialysis

## Abstract

***Summary*:**

Femoral artery calcification (FAC) is a significant predictor of hip fractures in hemodialysis patients. A higher FAC score is associated with increased fracture risk and poor survival outcomes. Identifying FAC through radiographic assessment may improve fracture risk stratification and clinical management in this high-risk population.

**Purpose:**

Patients with end-stage renal disease (ESRD) on hemodialysis (HD) are at increased risk for vascular calcification (VC) and bone fractures. While previous studies have linked aortic calcification with hip fractures, the relationship between medium-caliber artery-femoral artery calcification (FAC) and fall-related hip fractures in HD patients remains unclear.

**Methods:**

We retrospectively analyzed 170 HD patients who experienced falls and sought treatment in the emergency department (ED) between 2007 and 2014. The FAC score, representing the severity of femoral artery calcification, was calculated as the ratio of the total length of calcification plaques to the length of the femoral vessel visible on plain radiographs of the hip and femur. A logistic regression model assessed the association between FAC score and hip fracture risk, and receiver operating characteristic curve analysis evaluated its predictive power.

**Results:**

Among the 130 patients meeting inclusion criteria, 55 had fall-related hip fractures. The incidence rate of hip fractures among dialysis patients was 6.18 cases per 1000 person-years by dividing the total number of hip fracture events by the cumulative dialysis duration (in years) of all enrolled patients. Fracture patients were older and had lower serum creatinine, sodium, and albumin levels but higher aspartate aminotransferase levels. The fracture group also had a higher FAC score (0.47 [IQR, 0.28 – 0.76] vs. 0.00 [IQR, 0.00 – 0.40], *p* < 0.001). Multivariable analysis identified old age, heart failure with reduced ejection fraction (EF), and higher FAC scores as independent risk factors for hip fractures. Survival curves showed increased mortality among patients with higher FAC scores and hip fractures (*p* < 0.01). Conclusion.

High FAC scores were associated with an increased risk of hip fractures in HD patients, independent of traditional risk factors, and were linked to poor survival outcomes.

**Supplementary Information:**

The online version contains supplementary material available at 10.1007/s11657-025-01536-1.

## Introduction

Chronic kidney disease-mineral bone disorder (CKD-MBD) is a prevalent complication among CKD patients, especially those progressing to end-stage renal disease (ESRD) requiring dialysis. This condition encompasses a spectrum of clinical presentations involving vascular calcification (VC) and bone abnormalities, both of which increase the risk of fracture in CKD patients compared to the general population [1–3]. As expected, hip fractures in CKD patients are associated with substantial healthcare costs and increased mortality [4], underscoring the necessity to identify at-risk individuals and prevent fractures in this vulnerable population.

Emerging studies indicate that CKD patients share similar fracture risk factors with the general population but exhibit unique characteristics such as abnormal serum Pi levels, hyperparathyroidism, vitamin D deficiency, and elevated FGF-23 levels [5, 6]. VC, a hallmark of CKD-MBD, is associated with cardiovascular morbidity and mortality in ESRD patients and has been linked to an increased risk of fractures [7, 8]. Meta-analyses and observational studies in the general population have shown significant correlations between aortic calcification and skeletal fractures, as well as between coronary artery calcification (CAC) and hip fractures [9–12]. However, few studies have examined the link between VC and the risk of fractures in ESRD patients [13, 14]. One prospective study found aortic arch calcification predicted major fractures (hip, pelvis, humerus, proximal forearm, lower leg, or vertebrae) in dialysis patients [14], while another study observed a strong association between vertebral fractures and VC in medium-caliber arteries (femoral, uterine/spermatic, and radial) but no correlation with non-vertebral osteoporotic fractures [15]. Currently, the relationship between medium-caliber arterial VC and non-vertebral fractures remains under investigation.

In this study, we aimed to explore whether there is an association between femoral artery calcification (FAC) and the risk of hip fracture in hemodialysis (HD) patients. A simple calcification score of the femoral artery, which is the ratio of the total length of calcification plaques to the vessel length on plain radiographs of the hip and femur, was used to quantify FAC severity. Our study demonstrated that the FAC score predicts HD patients’ hip fracture risk independent of traditional risk factors.

## Methods

### Patient enrollment and group assignment

This study was performed at a single medical center in Northern Taiwan. The dialysis patients participating in this study consistently underwent maintenance HD at the same hospital. We conducted a retrospective analysis of the medical records of 170 HD patients who sustained falls and received treatment in the emergency department (ED) from January 2007 to December 2014. Plain X-rays of the pelvis or femur were employed to identify hip fractures, and only patients with femoral neck fractures and intertrochanteric hip fractures resulting from these falls were evaluated. All fractures analyzed in the study were low-trauma injuries, typically caused by falls from standing height or lower. Based on pelvic or hip radiographs, the HD patients were categorized into groups with hip and no hip fractures. This study aimed to investigate the risk factors for and impacts of FAC on hip fractures and the long-term clinical outcomes for up to 10 years after hip fractures in HD patients. This study was conducted in compliance with the Declaration of Helsinki. Data were collected from electronic medical records, and the study was approved by the Institutional Review Board of the Tri-Service General Hospital (TSGH), National Defense Medical Center, Taiwan (TSGHIRB No.: A202305066).

### Inclusion and exclusion criteria

**Fig. 1:**
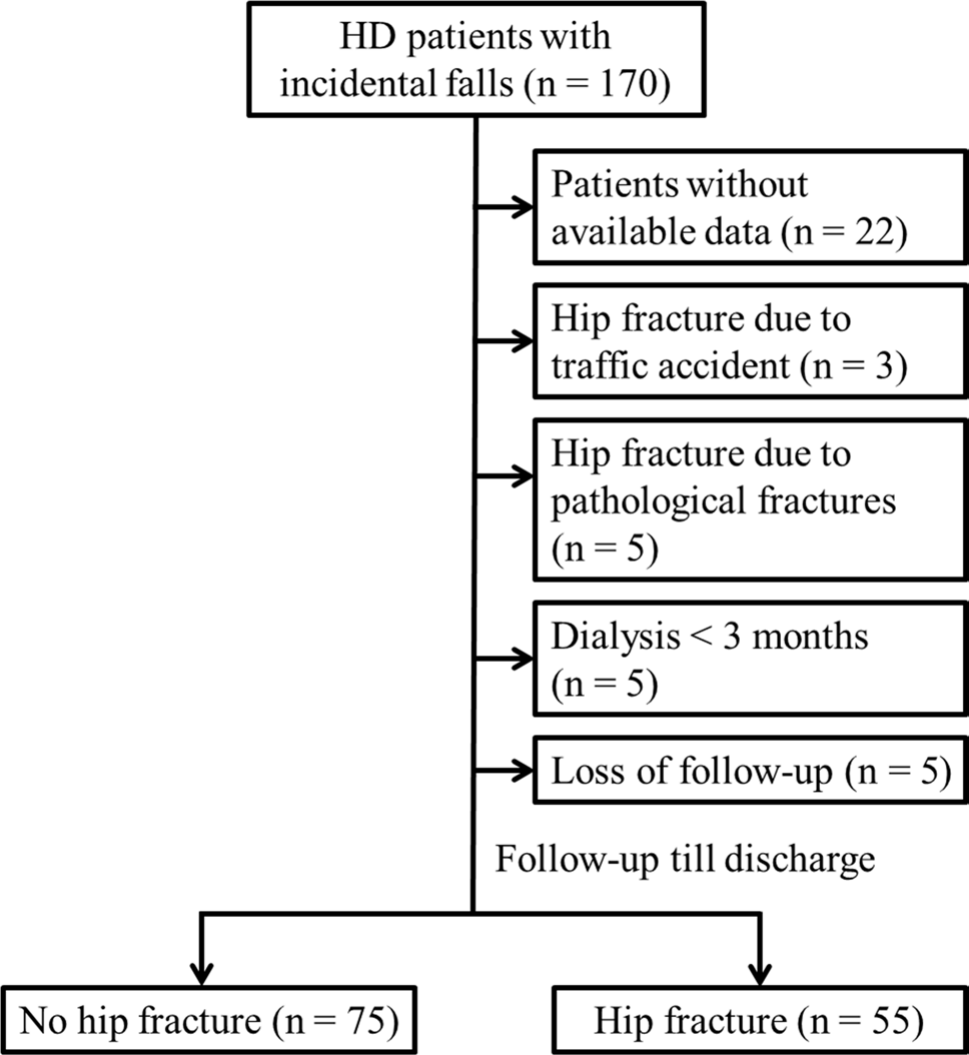
Flowchart depicting the selection process of hemodialysis patients without and with hip fractures

The inclusion criteria were as follows: (i) 20 years of age or older; (ii) ESRD on maintenance HD; (iii) a radiograph diagnosis with and without hip fracture; (iv) available clinical and laboratory data. The exclusion criteria were as follows: (i) fractures due to high-energy trauma (e.g., traffic accidents or falls from heights); (ii) pathological fractures caused by underlying malignancies, primary bone disorders, and metabolic bone diseases other than CKD-MBD; (iii) previous history of hip surgery; (iv) on HD for less than 3 months. A total of 130 patients with available demographics and laboratory data were enrolled (Fig. 1).

### Demographic data and laboratory parameters

All relevant clinical data, including gender, age, duration of dialysis, comorbidities, and echocardiography data, were extracted from the medical records of all eligible subjects. The laboratory data collected during patient check-ups and admission for incident hip fractures included hemoglobin (Hb), serum creatinine (Cr), blood urea nitrogen (BUN), sodium (Na), potassium (K), total calcium (Ca), phosphorus (P), alkaline phosphatase (ALP), intact parathyroid hormone (iPTH), aspartate aminotransferase (AST), alanine aminotransferase (ALT), and albumin levels. These parameters were measured by standard laboratory techniques using an automatic analyzer. Ejection fraction (EF) was assessed via transthoracic echocardiography during routine outpatient evaluations or hospital admissions. EF data were collected in the same year that the HD patients suffered falls. The treatment of hip fracture included intramedullary nailing, hemiarthroplasty, dynamic hip screw, or conservative therapies, and the choice of surgery depends on factors such as age, functional level, bone quality, and fracture type. All follow-up data and survival outcomes were acquired by a retrospective chart review during the study period.

### Measurement of FAC scores in the hip region

**Fig. 2:**
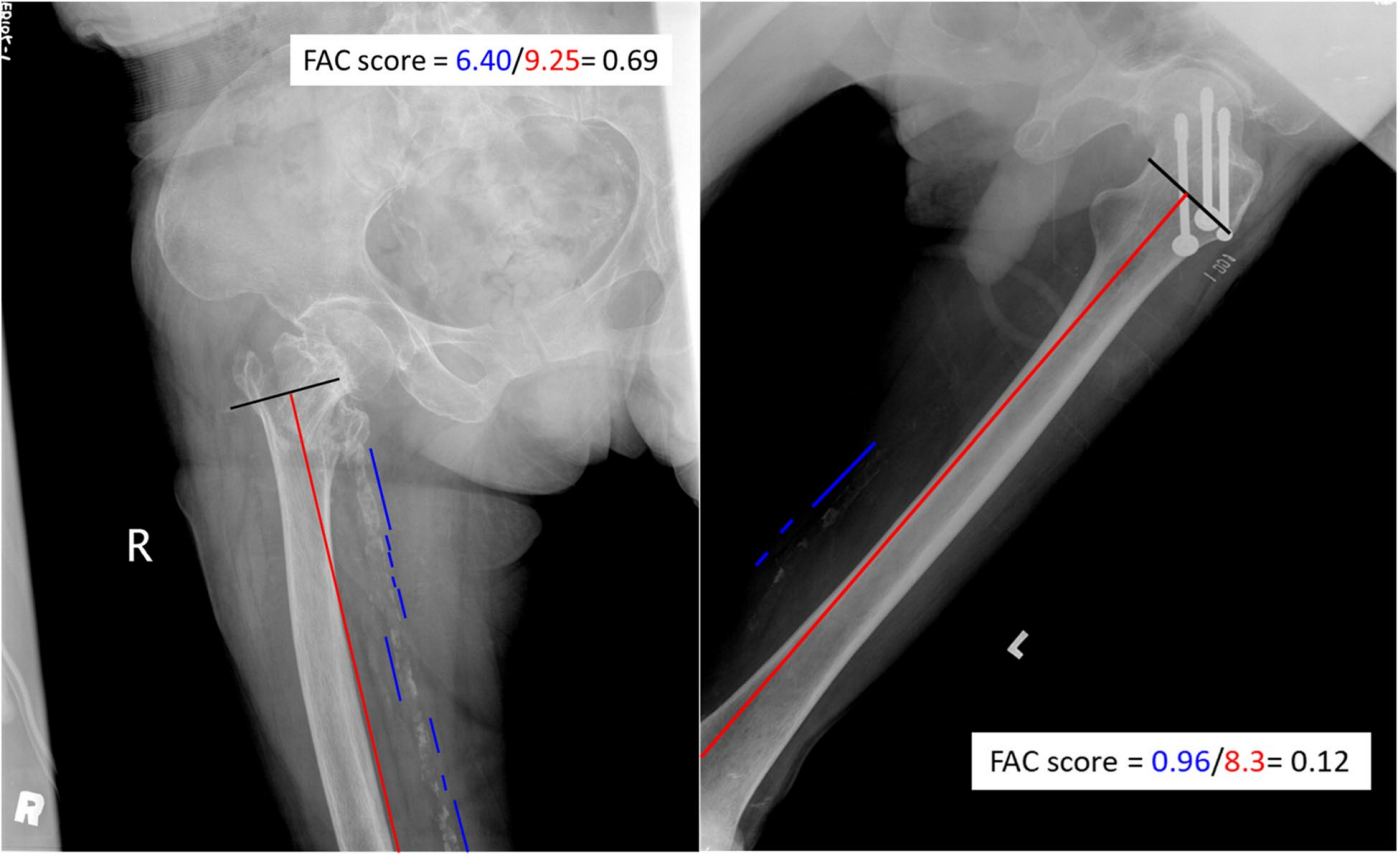
A representative radiographic image illustrates the measurement of the FAC score. The FAC score was calculated as the total length of calcification plaques divided by the length of the femoral vessel seen on plain radiographs

All patients underwent posterior-anterior and lateral X-rays of the hip at the ED. These X-rays were interpreted by orthopedic physicians consulted in the ED, and all images were adequately qualified and of sufficient length for further assessment of the FAC score. The FAC score, representing the severity of femoral artery calcification, was calculated as the ratio of the total length of calcification plaques to the length of the femoral vessel visible on plain radiographs of the hip and femur. The femoral arteries were chosen for scoring because these arteries supplied the femoral head, intertrochanteric territory, and femoral diaphyseal shaft via the branched femoral nutrient artery (FNA) [16]. FAC scores were calculated using a standardized protocol and verified by another trained clinician to minimize intra- and inter-reader variability. The measurement was performed using the ImageJ software (Fiji package), as illustrated in Fig. 2. The FAC scores were expressed as the median and interquartile range (IQR), and the receiver operating characteristic (ROC) curve analysis was employed to determine the most predictive threshold for the FAC score. The threshold was established by identifying the value that maximized sensitivity and specificity, resulting in the maximum area under the curve (AUC).

### Statistical analyses

Data are presented as means ± standard deviation (SD) and percentages (%). Differences in study variables were analyzed using two-tailed unpaired Student’s *t*-tests or chi-square tests. Logistic regression models were used to estimate odds ratios (ORs) and 95% confidence intervals (CIs) to assess the risk factors associated with hip fractures. Variables with *p*-values < 0.05 in the multivariate analysis were considered independent risk factors. The optimal threshold value for the FAC score was determined using the Youden index, a standard method for maximizing sensitivity and specificity. The ROC curve analysis identified 0.27 as the most predictive threshold for distinguishing patients at higher risk of hip fractures. Additionally, a ROC curve analysis was conducted to evaluate the predictive ability of each identified variable for hip fractures in HD patients. A retrospective Kaplan–Meier survival analysis was conducted to assess all-cause and cardiovascular mortality in patients with elevated FAC scores or hip fractures. Hazard ratios for survival were calculated by the Cox proportional hazards model. Statistical analysis was performed using IBM SPSS Statistics version 29.0, and significance was set at a* p*-value < 0.05.

## Results

### Baseline demographic characteristics in HD patients with and without fractures

A total of 130 HD patients who met the inclusion criteria were enrolled in the study. The study cohort consisted of 69 female participants (53%) and 61 male participants (47%), with an average age of 69.6 years (range 49 – 92). Among these patients, 75 individuals (58%), 95 individuals (74%), and 17 individuals (13%) were diagnosed with diabetes, hypertension, and cancer, respectively. Prior to enrollment, the average length of dialysis was 4.5 years, with a range of 0.4 to 27.0 years. FAC was present in around half of the study population (67/130, 52%). Ten percent of them (13/130, 10%) had an intertrochanteric hip fracture, while a third (42/130, 32%) suffered a femur neck fracture. The incidence rate of hip fractures among dialysis patients was calculated as 6.18 cases per 1000 person-years. The fracture group exhibited a higher proportion of females (69% vs. 41%, *p* < 0.01) and an advanced age (75.04 vs. 65.06 years, *p* < 0.0001) compared to the no-fracture group. However, no statistically significant differences were observed in the occurrence of chronic comorbidities or the duration of dialysis treatment between the two groups (Table 1).

**Table 1:** Comparison of baseline demographics, clinical features, and laboratory data between no-hip fracture and hip fracture groups

Variable	No-fracture group	Fracture group	*p-*value
	*n* = 75	*n* = 55	
Patient characteristics			
Gender			< 0.01
Men, *n* (%)	44 (59)	17 (31)	
Women, *n* (%)	31 (41)	38 (69)	
Age, years	65.56 ± 13.38	75.04 ± 9.28	< 0.0001
Duration on dialysis, years	4.56 ± 3.32	4.38 ± 5.46	NS
Hypertension, %	80	66	NS
Diabetes, %	57	58	NS
Cancer, %	9	18	NS
Echocardiography			
EF, %	62 ± 10	55 ± 14	< 0.01
PAP, mmHg	37.32 ± 17.64	40.00 ± 17.07	NS
FAC measurement			
FAC score (IQR)	0.00 (0.00 – 0.40)	0.47 (0.28 – 0.76)	< 0.001
Laboratory data			
Cr (mg/dL)	8.60 ± 3.27	6.09 ± 2.90	< 0.0001
Na (mEq/L)	137.31 ± 2.96	135.89 ± 4.23	< 0.05
Ca (mg/dL)	9.26 ± 0.92	9.00 ± 1.04	NS
P (mg/dL)	4.96 ± 1.64	4.18 ± 1.75	NS
iPTH (pg/ml)	438.62 ± 427.75	291.21 ± 250.82	NS
ALP (U/L)	103.48 ± 98.38	91.34 ± 48.97	NS
AST (U/L)	20.25 ± 11.96	26.42 ± 14.80	< 0.05
Albumin (g/dL)	3.79 ± 0.52	3.49 ± 0.59	< 0.001

*EF* ejection fraction, *PAP* pulmonary artery pressure, *FAC* femoral artery calcification, *Cr* creatinine, *Na* sodium, *Ca* calcium, *P* phosphorus, *iPTH* intact parathyroid hormone, *ALP* alkaline phosphatase, *AST* aspartate aminotransferase

### Comparative analysis of FAC scores and laboratory parameters

As shown in Table 1, the FAC score (0.47 [IQR, 0.28 – 0.76] vs. 0.00 [IQR, 0.00 – 0.40], *p* < 0.001) and serum AST level (26.42 vs. 20.25 U/L, *p* < 0.05) were significantly higher in the fracture group as compared to the no-fracture group. In contrast, the fracture group had a notable reduction in systolic ejection fraction ([EF], 55 vs. 62%, *p* < 0.01), along with decreased levels of serum Cr (6.09 vs. 8.60 mg/dL, *p* < 0.0001), Na (135.89 vs. 137.31 mEq/L, *p* < 0.05), and albumin (3.49 vs. 3.79 g/dL, *p* < 0.001). While there was no significant difference in the prevalence of diabetes between the fracture and no-fracture groups, it was observed that fracture patients with diabetes had the highest FAC score compared to both fracture patients without diabetes and no-fracture patients with or without diabetes (Supplementary Fig. 1).

### Univariate and multivariate analysis on predictors for hip fracture in HD patients

Univariate analysis showed that female gender, age, EF, FAC score, Cr, Na, AST, and albumin were significantly associated with hip fracture. In the multivariate analysis, age (odds ratio [OR] per year, 1.116; 95% confidence interval [CI], 1.034 – 1.204, *p* < 0.01), EF (OR per 1% increase, 0.946; 95% CI, 0.898 – 0.997, *p* < 0.05), and FAC score (OR per 0.1 increment, 1.243; 95% CI, 1.015 – 1.524, *p* < 0.05) were identified as independent predictors of hip fractures (Table 2).

**Table 2:** Univariate and multivariate logistic regression analysis of risk factors for incidental hip fractures

Variable	Univariable			Multivariable		
	OR	95% CI	***p***-value	OR	95% CI	*p*-value
Gender, female	3.173	1.523 – 6.608	< 0.01	2.307	0.603 – 8.832	NS
Age (year)	1.078	1.039 – 1.118	< 0.001	1.116	1.034 – 1.204	< 0.01
EF (%)	0.951	0.918 – 0.984	< 0.01	0.946	0.898 – 0.997	< 0.05
FAC score (per 0.1)	1.216	1.083 – 1.366	< 0.001	1.243	1.015 – 1.524	< 0.05
Cr (mg/dL)	0.765	0.671 – 0.873	< 0.001	0.819	0.619 – 1.085	NS
Na (mEq/L)	0.893	0.806 – 0.989	< 0.05	0.903	0.755 – 1.080	NS
AST (U/L)	1.039	1.008 – 1.070	< 0.05	1.015	0.972 – 1.060	NS
Albumin (g/dL)	0.381	0.184 – 0.788	< 0.01	0.483	0.140 – 1.667	NS

^*^The FAC score is unitless, calculated as the ratio of the total length of calcification plaques to the length of the femoral vessel visible on plain radiographs

**Fig. 3:**
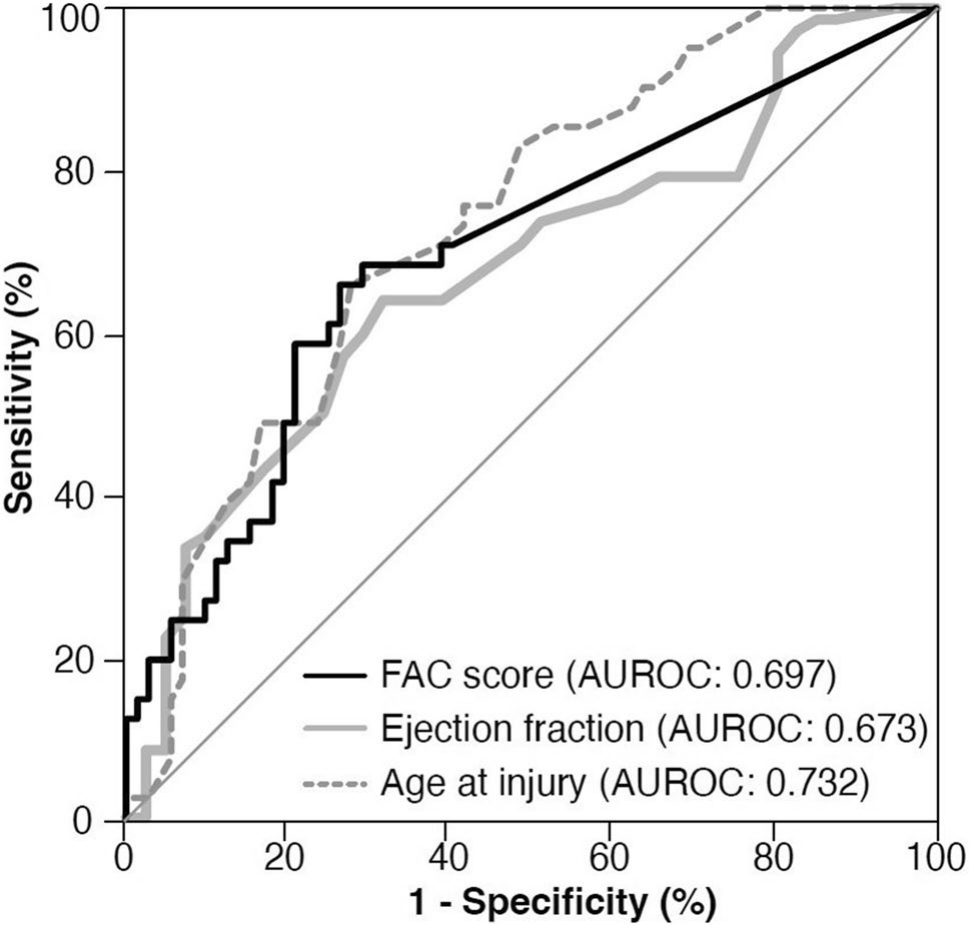
ROC curve analysis for the prediction of a hip fracture. ROC curves using three different parameters to predict hip fractures. The AUC for each color represents the overall performance of that parameter in predicting hip fractures

**Fig. 4:**
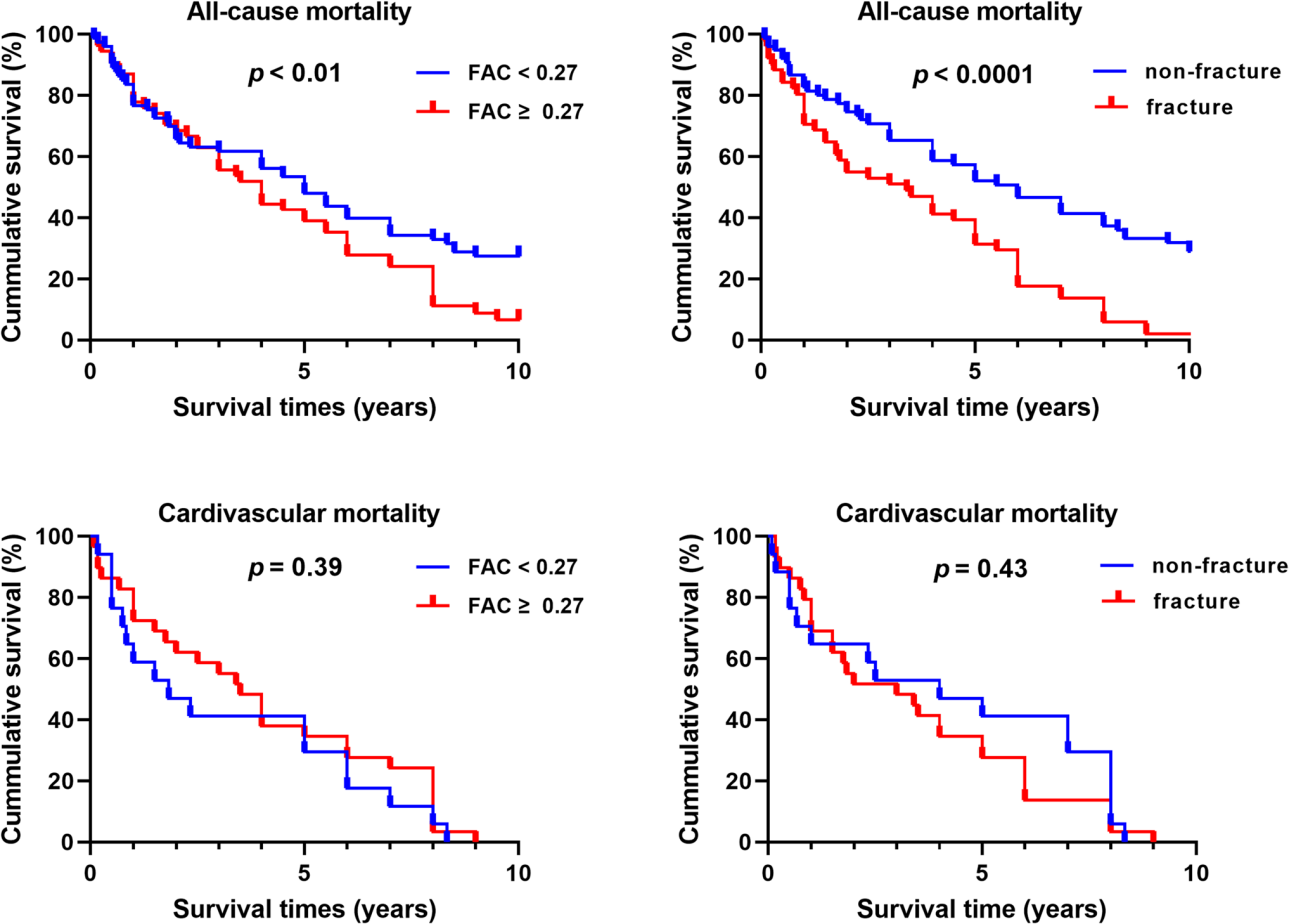
Kaplan–Meier survival curves for all-cause and cardiovascular mortality in hemodialysis patients. Patients with FAC scores ≥ 0.27 had significantly lower cumulative survival for all-cause mortality (*p* < 0.01), but no significant difference was observed for cardiovascular mortality. Fracture patients showed significantly lower cumulative survival for all-cause mortality (*p* < 0.0001), while no significant difference was observed for cardiovascular mortality (*p* = 0.43)

The predictive performance of age, EF, and FAC score for hip fracture was assessed using ROC analysis. The resulting AUC values for age, EF, and FAC score were 0.732, 0.673, and 0.697, respectively. The cutoff value of 0.27 for the FAC score, identified through ROC curve analysis, provides a clinically meaningful threshold for stratifying hip fracture risk in HD patients, with a sensitivity of 75% and specificity of 82% (Fig. 3). Patients were subsequently categorized based on the cutoff value of the FAC score (0.27). Patients with FAC scores ≥ 0.27 had a significantly higher risk of hip fractures compared to those with FAC scores < 0.27. The OR for hip fractures in individuals with FAC ≥ 0.27 was 3.391 (95% CI: 1.635 – 7.032, *p* < 0.05). There were significant differences in the prevalence of diabetes (43 vs. 77%, *p* < 0.0001) and serum total calcium levels (9.10 mg/dL vs. 9.39 mg/dL, *p* < 0.05) among individuals with low and high FAC scores (Supplementary Table 1). Patients with a low FAC score (< 0.27) had a median survival time of 5 years, while those with a high FAC score (≥ 0.27) had a median survival time of 4 years (*p* < 0.01). The Kaplan–Meier survival analysis revealed a higher HR of 1.57 (95% CI: 1.04 – 2.37, *p* < 0.01, Fig. 4) for increased overall mortality in those with higher FAC scores. However, there was no statistically significant association between FAC score and cardiovascular mortality (HR, 95% CI: 0.29 – 0.95, *p* = 0.39). The median survival time for fracture and no-fracture patients after hip fracture was 3.4 and 6.0 years, respectively. Fracture patients had a twofold higher mortality risk than their no-fracture counterparts (HR 2.48, 95% CI: 1.60 – 3.85, *p* < 0.0001, Fig. 4).

## Discussion

This study represents the first to investigate the relationship between FAC and hip fractures in HD patients. We identified a hip fracture incidence rate of 6.18 cases per 1000 person-years among dialysis patients, a rate consistent with previously reported findings [3, 17, 18]. Risk factors for hip fractures included female gender, advanced age, heart failure, FAC scores, and malnutrition. The multivariate-adjusted analyses revealed advanced age, reduced EF, and higher FAC scores as independent predictors of hip fracture risk. Additionally, both higher FAC scores and hip fractures exhibited independent correlations with poorer overall survival outcomes.

FAC, a common manifestation of VC, affected 40–70% of advanced CKD and dialysis patients. In this study, we established a significant association between FAC scores and hip fractures, even after controlling for confounding variables [19, 20]. Our results indicate that calcification in the abdominal aorta, carotid artery, coronary artery, and peripheral artery calcification (PAC) is associated with health outcomes [21]. In line with our observation, Szulc et al. reported that severe PAC is associated with a higher risk of acute coronary syndrome and major adverse cardiovascular events in older men [22]. Furthermore, HD patients with iliac artery calcifications are at high risk of vertebral fractures [23]. The mechanisms linking FAC and hip fractures remain incompletely elucidated; however, they likely involve ischemia-induced weakening of bone structure, contributing to diminished bone density by disrupting essential metabolic processes, including nutrient transport, waste removal, tissue repair, and bone remodeling [16, 24, 25]. These effects, compounded by systemic factors such as secondary hyperparathyroidism, mineral dysregulation, and malnutrition, can weaken bone integrity and elevate fracture risk [26, 27]. Additionally, previous studies have highlighted that vascular calcification, through its association with CKD-MBD, significantly alters bone metabolism and quality, as evidenced by localized mineralized bone loss in dialysis patients and its histomorphometric appraisal [28, 29]. This multifactorial interaction underscores the complexity of vascular and skeletal abnormalities in this patient population.

VC is a component of CKD-MBD, associated with disturbances in Pi and Ca homeostasis, as well as mineralotropic hormones like PTH, FGF23, and 1,25-dihydroxyvitamin D_3_ (1,25(OH)_2_D_3_). In addition to ischemia caused by VC, disturbed mineral metabolism in CKD patients may contribute to increased fracture susceptibility [30, 31]. Phosphorus accumulation begins in CKD Stage 3b, with hyperphosphatemia firmly linked to VC. High Pi exposure in vitro induces osteogenic transformation of vascular smooth muscle cells (VSMCs) via RUNX2 overexpression and mineral deposition [32]. Conversely, the knockdown of type III sodium‐dependent phosphate co-transporters (PiT 1) inhibits calcification and osteogenic markers. High Pi also stimulates matrix vesicle (MV) synthesis, oxidative stress, and mitochondrial dysfunction, all contributing to progressive VC [32]. Dialysis patients often develop hypercalcemia from high-Ca dialysate, vitamin D therapy, Ca-based Pi binders, and tertiary hyperparathyroidism, further promoting MV-mediated mineralization of VSMCs [33]. However, our study found comparable serum Pi and Ca levels in fracture and no-fracture groups, suggesting additional factors at play.

FGF23 and PTH decrease renal Pi reabsorption by downregulating the type IIa sodium-phosphate co-transporter expression in proximal tubules [34]. Previous studies have demonstrated that both hormones are associated with VC and fracture risk in CKD patients. The Longitudinal Aging Study Amsterdam reported a higher cardiovascular risk and abdominal aortic calcifications (AAC) in men with elevated serum PTH [35]. Another clinical trial found that nondialysis CKD patients exhibiting elevated AAC or pelvic arterial calcification scores presented with increased serum Pi and PTH [36]. In contrast, the role of increased FGF23 in VC progression is debatable. While some studies associate elevated FGF23 with aortic calcification [37, 38], others, such as the Chronic Renal Insufficiency Cohort (CRIC) study, found no association with CAC [39]. These clinical discrepancies are supported by some in vitro experiments. PTH-induced VSMC calcification involves apoptosis and endoplasmic reticulum stress via C/EBP Homologous Protein (CHOP) and Jun-N-terminal kinase (JNK) signaling [40]. In contrast, FGF23 may inhibit calcification through Wnt7b/β-catenin modulation [41]. Collectively, elevated PTH likely contributes to VC, heightening fracture risk; however, comparable PTH levels between fracture and non-fracture groups in our study suggest a negligible role of PTH in FAC and hip fractures.

Malnutrition is linked to frailty fractures and contributes to VC and bone fractures through the malnutrition-inflammation complex syndrome (MICS), which is prevalent in CKD and chronic heart failure (CHF). MICS affects 40–60% of HD patients, particularly those with elevated C-reactive protein (CRP) levels [42, 43]. Similarly, CHF patients exhibit high rates of inflammation and malnutrition [44–46]. Antioxidant deficiencies in malnourished CKD patients promote oxidative stress and inflammation, while proinflammatory cytokines accelerate protein degradation, malnutrition, and VC [47, 48]. In uremic rats, low dietary protein raised serum osteocalcin, a marker of high bone turnover and augmented arterial calcification [49]. Inflammatory cytokines (TNF-α, IL-1β, IL-6) initiate VC by upregulating BMP2 and downregulating SMα-actin expression [50–52]. These cytokines also accelerate bone resorption, linking MICS to fractures [53–56]. Consistent with these findings, we observed lower EF and serum albumin levels in HD patients with hip fractures and identified reduced EF as an independent fracture risk factor. These findings suggest that inflammations induced by lower EF and malnutrition are significant contributors to VC and bone fractures.

As VC is linked to increased cardiovascular events and mortality [15, 57], the Kidney Disease Improving Global Outcome (KDIGO) study group recommends VC screening in CKD patients using lateral abdominal radiography or CT [58]. Plain X-rays are a simple, widely used tool, with various grading systems predicting adverse CKD outcomes [59–61]. In this study, we introduced a FAC scoring system based on calcification plaque length relative to vessel length in plain hip radiographs. This score significantly differed between the fracture and no-fracture groups, correlating with increased fracture risk and mortality. Kaplan–Meier survival analysis revealed a mortality HR of 1.57 for patients with higher FAC scores (> 0.27), aligning with prior studies [36, 57, 62, 63]. These findings suggest that FAC is an important factor for hip fractures in dialysis patients, warranting further research to confirm and clarify underlying mechanisms.

## Limitations

This study has several limitations that should be acknowledged. First, its retrospective nature and the relatively small sample size from a single medical institution may introduce biases and uncontrolled confounding variables. Second, we did not assess proinflammatory cytokines or hormones such as FGF23 and 25-hydroxyvitamin D (25-OHD) levels, which are known to influence VC and fracture risk. Third, the lack of detailed treatment data regarding the use of active vitamin D analogs or calcimimetics and the absence of bone biopsies restrict our ability to determine the precise bone turnover status or the specific causes of fractures. Fourth, we only calculated FAC scores and did not analyze calcification in other major arteries, assess sarcopenia status, or account for patients’ medications, which may affect hip fracture events in vulnerable dialysis patients. Fifth, the FAC score is significantly correlated with overall mortality, underscoring its clinical relevance. The insignificance of cardiovascular mortality may be attributed to several factors, such as the limited sample size for cardiovascular death events and the complex nature of mortality among dialysis patients. Sixth, the performance of FAC may restrict its efficacy as a standalone predictor and incorporating additional markers or composite models is essential to improve predictive accuracy.

## Conclusion

In summary, our findings suggest that the FAC score is a key factor linked to an increased risk of hip fractures and mortality in dialysis patients. Further investigations are warranted to elucidate the underlying mechanisms and explore whether interventions targeting FAC could enhance bone health and overall outcomes in this high-risk population.

## Supplementary Information

Below is the link to the electronic supplementary material.

ESM 1 (PNG 903 KB)

High Resolution Image (TIF 5.97 MB)

ESM 2 (DOCX 15.4 KB)

## Data Availability

The data generated and analyzed in this study are presented in the manuscript. The raw data supporting the conclusions of this article are available from the corresponding author upon reasonable request.

## References

[1] Wu PY, Chen SC, Lin YC et al (2022) Role of fracture risk assessment tool and bone turnover markers in predicting all-cause and cardiovascular mortality in hemodialysis patients. Front Med (Lausanne) 9:891363 35463031 10.3389/fmed.2022.891363 PMC9021425

[2] Slouma M, Sahli H, Bahlous A et al (2020) Mineral bone disorder and osteoporosis in hemodialysis patients. Adv Rheumatol 60:15 32102689 10.1186/s42358-020-0118-0

[3] Alem AM, Sherrard DJ, Gillen DL, Weiss NS, Beresford SA, Heckbert SR, Wong C, Stehman-Breen C (2000) Increased risk of hip fracture among patients with end-stage renal disease. Kidney Int 58:396–399 10886587 10.1046/j.1523-1755.2000.00178.x

[4] Perez-Saez MJ, Prieto-Alhambra D, Barrios C, Crespo M, Redondo D, Nogues X, Diez-Perez A, Pascual J (2015) Increased hip fracture and mortality in chronic kidney disease individuals: the importance of competing risks. Bone 73:154–159 25549867 10.1016/j.bone.2014.12.020

[5] Pimentel A, Ureña-Torres P, Bover J, Luis Fernandez-Martín J, Cohen-Solal M (2021) Bone fragility fractures in CKD patients. Calcif Tissue Int 108:539–550 33219822 10.1007/s00223-020-00779-z PMC8052229

[6] Przedlacki J, Buczyńska-Chyl J, Koźmiński P et al (2018) The utility of FRAX® in predicting bone fractures in patients with chronic kidney disease on hemodialysis: a two-year prospective multicenter cohort study. Osteoporos Int J Established Result Cooperation Eur Found Osteoporos Natl Osteoporos Found USA 29:1105–1115 10.1007/s00198-018-4406-z 29411069

[7] Moe SM (2006) Vascular calcification and renal osteodystrophy relationship in chronic kidney disease. Eur J Clin Invest 36(Suppl 2):51–62 16884398 10.1111/j.1365-2362.2006.01665.x

[8] Moe SM, Chen NX (2008) Mechanisms of vascular calcification in chronic kidney disease. J Am Soc Nephrol 19:213–216 18094365 10.1681/ASN.2007080854

[9] Chen Z, Yu Y (2016) Aortic calcification was associated with risk of fractures: a meta-analysis. J Back Musculoskelet Rehabil 29:635–642 27232081 10.3233/BMR-160700

[10] Wei D, Zheng G, Gao Y, Guo J, Zhang T (2018) Abdominal aortic calcification and the risk of bone fractures: a meta-analysis of prospective cohort studies. J Bone Miner Metab 36:439–446 28642974 10.1007/s00774-017-0849-0

[11] Chuang TL, Li YD, Hsiao FT, Chuang MH, Wang YF (2017) FRAX® fracture risks are associated with coronary artery calcification score. Dis Markers 2017:1592598 29422704 10.1155/2017/1592598 PMC5750485

[12] Bjelobrk MB, Kovacevic MK, Cankovic MC et al (2023) Impact of coronary artery calcium score level in prediction of major adverse cardiovascular events. Eur Heart J 44(ehad655):1267

[13] Jafari M, Anwar S, Kour K, Sanjoy S, Goyal K, Prasad B (2021) T Scores, FRAX, Frailty phenotype, falls, and its relationship to fractures in patients on maintenance hemodialysis. Can J Kidney Health Dis 8:20543581211041184 34457317 10.1177/20543581211041184 PMC8392815

[14] Chen HY, Chiu YL, Hsu SP, Pai MF, Yang JY, Peng YS (2016) Relationship between fetuin A, vascular calcification and fracture risk in dialysis patients. PLoS ONE 11:e0158789 27398932 10.1371/journal.pone.0158789 PMC4939952

[15] Rodríguez-García M, Gómez-Alonso C, Naves-Díaz M, Diaz-Lopez JB, Diaz-Corte C, Cannata-Andía JB (2009) Vascular calcifications, vertebral fractures and mortality in haemodialysis patients. Nephrol Dial Transplant Off Publ Eur Dial Transplant Assoc Eur Ren Assoc 24:239–246 10.1093/ndt/gfn466 PMC2639312 18725376

[16] Kawasaki Y, Kinose S, Kato K, Sakai T, Ichimura K (2020) Anatomic characterization of the femoral nutrient artery: application to fracture and surgery of the femur. Clin Anat 33:479–487 31008535 10.1002/ca.23390

[17] Tan J, Li Y, Wu Z, Zhao J (2018) Risk of hip fracture in patients on dialysis or kidney transplant: a meta-analysis of 14 cohort studies. Ther Clin Risk Manag 14:1747–1755 30288044 10.2147/TCRM.S171970 PMC6159787

[18] Hickson LJ, Farah WH, Johnson RL, Thorsteinsdottir B, Ubl DS, Yuan BJ, Albright R, Rule AD, Habermann EB (2018) Death and postoperative complications after hip fracture repair: dialysis effect. Kidney Int Rep 3:1294–1303 30450456 10.1016/j.ekir.2018.07.001 PMC6224855

[19] Toussaint ND, Lau KK, Strauss BJ, Polkinghorne KR, Kerr PG (2008) Associations between vascular calcification, arterial stiffness and bone mineral density in chronic kidney disease. Nephrol Dial Transplant 23:586–593 17933842 10.1093/ndt/gfm660

[20] Choi NG, Choi BY, DiNitto DM, Marti CN, Kunik ME (2019) Fall-related emergency department visits and hospitalizations among community-dwelling older adults: examination of health problems and injury characteristics. BMC Geriatr 19:303 31711437 10.1186/s12877-019-1329-2 PMC6849272

[21] Laclaustra M, Casasnovas JA, Fernández-Ortiz A et al (2016) Femoral and carotid subclinical atherosclerosis association with risk factors and coronary calcium: the AWHS Study. J Am Coll Cardiol 67:1263–1274 26988945 10.1016/j.jacc.2015.12.056

[22] Szulc P, Planckaert C, Foesser D, Patsch J, Chapurlat R (2021) High cardiovascular risk in older men with severe peripheral artery calcification on high-resolution peripheral QCT scans: The STRAMBO Study. Arterioscler Thromb Vasc Biol 41:1818–1829 33792348 10.1161/ATVBAHA.120.315289

[23] Fusaro M, Tripepi G, Plebani M et al (2021) The vessels-bone axis: iliac artery calcifications, vertebral fractures and vitamin K from VIKI study. Nutrients 13(10):3567. 10.3390/nu13103567 34684568 10.3390/nu13103567 PMC8539275

[24] Vogt MT, Cauley JA, Kuller LH, Nevitt MC (1997) Bone mineral density and blood flow to the lower extremities: the study of osteoporotic fractures. J Bone Miner Res 12:283–289 9041062 10.1359/jbmr.1997.12.2.283

[25] Ramasamy SK, Kusumbe AP, Schiller M et al (2016) Blood flow controls bone vascular function and osteogenesis. Nat Commun 7:13601 27922003 10.1038/ncomms13601 PMC5150650

[26] Kapitola J, Zák J (1998) Relation between local blood flow and bone mineralization indicators in the tibia of female rats. Sb Lek 99:349–354 10803274

[27] Birişik F, Bilgin Y, Bayram S, Öztürkmen Y (2021) Does presence of femoral arterial calcification have an effect on postoperative complication and mortality in patients with hip fracture? Cureus 13:e14878 34113507 10.7759/cureus.14878 PMC8177717

[28] Parfitt AM, Qiu S, Rao DS (2004) The mineralization index–a new approach to the histomorphometric appraisal of osteomalacia. Bone 35:320–325 15207773 10.1016/j.bone.2004.02.016

[29] Schober HC, Han ZH, Foldes AJ, Shih MS, Rao DS, Balena R, Parfitt AM (1998) Mineralized bone loss at different sites in dialysis patients: implications for prevention. J Am Soc Nephrol 9:1225–1233 9644632 10.1681/ASN.V971225

[30] Hou YC, Lu CL, Lu KC (2018) Mineral bone disorders in chronic kidney disease. Nephrology (Carlton) 23(Suppl 4):88–94 30298663 10.1111/nep.13457

[31] Zaimi M, Grapsa E (2024) Current therapeutic approach of chronic kidney disease-mineral and bone disorder. Ther Apher Dial Off Peer-Reviewed J Int Soc Apher, Jpn Soc Apher, Jpn S Dial Ther 28:671–689 10.1111/1744-9987.14177 38898685

[32] Ding N, Lv Y, Su H, Wang Z, Kong X, Zhen J, Lv Z, Wang R (2023) Vascular calcification in CKD: new insights into its mechanisms. J Cell Physiol 238:1160–1182 37269534 10.1002/jcp.31021

[33] Kapustin AN, Davies JD, Reynolds JL et al (2011) Calcium regulates key components of vascular smooth muscle cell-derived matrix vesicles to enhance mineralization. Circ Res 109:e1–12 21566214 10.1161/CIRCRESAHA.110.238808

[34] Blau JE, Collins MT (2015) The PTH-vitamin D-FGF23 axis. Rev Endocr Metab Disord 16:165–174 26296372 10.1007/s11154-015-9318-z

[35] Hoogendijk EO, Deeg DJ, Poppelaars J et al (2016) The Longitudinal Aging Study Amsterdam: cohort update 2016 and major findings. Eur J Epidemiol 31:927–945 27544533 10.1007/s10654-016-0192-0 PMC5010587

[36] Disthabanchong S, Vipattawat K, Phakdeekitcharoen B, Kitiyakara C, Sumethkul V (2018) Abdominal aorta and pelvic artery calcifications on plain radiographs may predict mortality in chronic kidney disease, hemodialysis and renal transplantation. Int Urol Nephrol 50:355–364 29236239 10.1007/s11255-017-1758-9

[37] Nasrallah MM, El-Shehaby AR, Salem MM, Osman NA, El Sheikh E, Sharaf El Din UA (2010) Fibroblast growth factor-23 (FGF-23) is independently correlated to aortic calcification in haemodialysis patients. Nephrology, dialysis, transplantation : official publication of the European Dialysis and Transplant Association - European Renal Association 25:2679–2685 20176609 10.1093/ndt/gfq089

[38] Desjardins L, Liabeuf S, Renard C, Lenglet A, Lemke HD, Choukroun G, Drueke TB, Massy ZA (2012) FGF23 is independently associated with vascular calcification but not bone mineral density in patients at various CKD stages. Osteoporosis international : a journal established as result of cooperation between the European Foundation for Osteoporosis and the National Osteoporosis Foundation of the USA 23:2017–2025 22109743 10.1007/s00198-011-1838-0

[39] Scialla JJ, Lau WL, Reilly MP et al (2013) Fibroblast growth factor 23 is not associated with and does not induce arterial calcification. Kidney Int 83:1159–1168 23389416 10.1038/ki.2013.3 PMC3672330

[40] Duang S, Zhang M, Liu C, Dong Q (2022) Parathyroid hormone-induced vascular smooth muscle cells calcification by endoplasmic reticulum stress. J Physiol Pharmacol 73(5). 10.26402/jpp.2022.5.03 10.26402/jpp.2022.5.03 36942806

[41] Chen YX, Huang C, Duan ZB, Xu CY, Chen Y (2019) Klotho/FGF23 axis mediates high phosphate-induced vascular calcification in vascular smooth muscle cells via Wnt7b/β-catenin pathway. Kaohsiung J Med Sci 35:393–400 31001900 10.1002/kjm2.12072 PMC11900703

[42] Kovesdy CP, Kopple JD, Kalantar-Zadeh K (2013) Management of protein-energy wasting in non-dialysis-dependent chronic kidney disease: reconciling low protein intake with nutritional therapy. Am J Clin Nutr 97:1163–1177 23636234 10.3945/ajcn.112.036418 PMC3652918

[43] Ikizler TA, Wingard RL, Harvell J, Shyr Y, Hakim RM (1999) Association of morbidity with markers of nutrition and inflammation in chronic hemodialysis patients: a prospective study. Kidney Int 55:1945–1951 10231458 10.1046/j.1523-1755.1999.00410.x

[44] Lv S, Ru S (2021) The prevalence of malnutrition and its effects on the all-cause mortality among patients with heart failure: a systematic review and meta-analysis. PLoS ONE 16:e0259300 34710169 10.1371/journal.pone.0259300 PMC8553374

[45] Redfield MM, Chen HH, Borlaug BA et al (2013) Effect of phosphodiesterase-5 inhibition on exercise capacity and clinical status in heart failure with preserved ejection fraction: a randomized clinical trial. JAMA 309:1268–1277 23478662 10.1001/jama.2013.2024 PMC3835156

[46] Pfisterer M, Buser P, Rickli H et al (2009) BNP-guided vs symptom-guided heart failure therapy: the Trial of Intensified vs Standard Medical Therapy in Elderly Patients With Congestive Heart Failure (TIME-CHF) randomized trial. JAMA 301:383–392 19176440 10.1001/jama.2009.2

[47] Kalantar-Zadeh K, Kopple JD, Deepak S, Block D, Block G (2002) Food intake characteristics of hemodialysis patients as obtained by food frequency questionnaire. J Ren Nutr 12:17–31 11823990 10.1053/jren.2002.29598

[48] McCarthy DO (2000) Tumor necrosis factor alpha and interleukin-6 have differential effects on food intake and gastric emptying in fasted rats. Res Nurs Health 23:222–228 10871537 10.1002/1098-240x(200006)23:3<222::aid-nur6>3.0.co;2-3

[49] Price PA, Roublick AM, Williamson MK (2006) Artery calcification in uremic rats is increased by a low protein diet and prevented by treatment with ibandronate. Kidney Int 70:1577–1583 16955099 10.1038/sj.ki.5001841

[50] Agharazii M, St-Louis R, Gautier-Bastien A, Ung RV, Mokas S, Larivière R, Richard DE (2015) Inflammatory cytokines and reactive oxygen species as mediators of chronic kidney disease-related vascular calcification. Am J Hypertens 28:746–755 25430697 10.1093/ajh/hpu225

[51] Westenfeld R, Jahnen-Dechent W, Ketteler M (2007) Vascular calcification and fetuin-A deficiency in chronic kidney disease. Trends Cardiovasc Med 17:124–128 17482094 10.1016/j.tcm.2007.02.005

[52] Yamada S, Tokumoto M, Tsuruya K, Tatsumoto N, Noguchi H, Kitazono T, Ooboshi H (2015) Fetuin-A decrease induced by a low-protein diet enhances vascular calcification in uremic rats with hyperphosphatemia. Am J Physiol Renal Physiol 309:F744–754 26180236 10.1152/ajprenal.00017.2015

[53] Yamada S, Arase H, Yoshida H, Kitamura H, Tokumoto M, Taniguchi M, Hirakata H, Tsuruya K, Nakano T, Kitazono T (2022) Malnutrition-inflammation complex syndrome and bone fractures and cardiovascular disease events in patients undergoing hemodialysis: the Q-Cohort study. Kidney Med 4:100408 35386605 10.1016/j.xkme.2022.100408 PMC8978069

[54] Yamada S, Taniguchi M, Tokumoto M, Yoshitomi R, Yoshida H, Tatsumoto N, Hirakata H, Fujimi S, Kitazono T, Tsuruya K (2017) Modified creatinine index and the risk of bone fracture in patients undergoing hemodialysis: the Q-Cohort study. Am J Kidney Dis 70:270–280 28450093 10.1053/j.ajkd.2017.01.052

[55] McLean RR (2009) Proinflammatory cytokines and osteoporosis. Curr Osteoporos Rep 7:134–139 19968917 10.1007/s11914-009-0023-2

[56] Cauley JA, Danielson ME, Boudreau RM, Forrest KY, Zmuda JM, Pahor M, Tylavsky FA, Cummings SR, Harris TB, Newman AB (2007) Inflammatory markers and incident fracture risk in older men and women: the Health Aging and Body Composition Study. Journal of bone and mineral research : the official journal of the American Society for Bone and Mineral Research 22:1088–1095 17419681 10.1359/jbmr.070409

[57] Bellasi A, Di Lullo L, Russo D, Ciarcia R, Magnocavallo M, Lavalle C, Ratti C, Fusaro M, Cozzolino M, Di Iorio BR (2021) Predictive value of measures of vascular calcification burden and progression for risk of death in incident to dialysis patients. J Clin Med 10(3):376. 10.3390/jcm10030376 10.3390/jcm10030376 PMC7863918 33498192

[58] KDIGO (2017) Clinical practice guideline update for the diagnosis, evaluation, prevention, and treatment of chronic kidney disease-mineral and bone disorder (CKD-MBD). Kidney Int Suppl 7:1–59 10.1016/j.kisu.2017.04.001 PMC6340919 30675420

[59] Chao CT, Yeh HY, Hung KY (2023) Chest radiography deep radiomics-enabled aortic arch calcification interpretation across different populations. iScience 26:106429 37009230 10.1016/j.isci.2023.106429 PMC10050631

[60] Hong D, Wu S, Pu L et al (2013) Abdominal aortic calcification is not superior over other vascular calcification in predicting mortality in hemodialysis patients: a retrospective observational study. BMC Nephrol 14:120 23738982 10.1186/1471-2369-14-120 PMC3691830

[61] Górriz JL, Molina P, Cerverón MJ et al (2015) Vascular calcification in patients with nondialysis CKD over 3 years. Clin J Am Soc Nephrol CJASN 10:654–666 25770175 10.2215/CJN.07450714 PMC4386255

[62] Ohtake T, Mitomo A, Yamano M et al (2023) Impact of arterial calcification of the lower limbs on long-term clinical outcomes in patients on hemodialysis. J Clin Med 12(4):1299. 10.3390/jcm12041299 10.3390/jcm12041299 PMC9967859 36835836

[63] Aly GSG, Kassem HH, Hashad A, Salem MA, Labib D, Baligh E (2020) Lower extremity arterial calcifications assessed by multislice CT as a correlate to coronary artery disease. Egyptian Journal of Radiology and Nuclear Medicine 51:54

