# Supplementary figures and images for: Femoral artery calcification predicts hip fracture in maintenance hemodialysis patients
Source: Arch Osteoporos. 2025 Aug 9;20(1):112. doi: 10.1007/s11657-025-01536-1 (PMC12334447; doi:10.1007/s11657-025-01536-1)

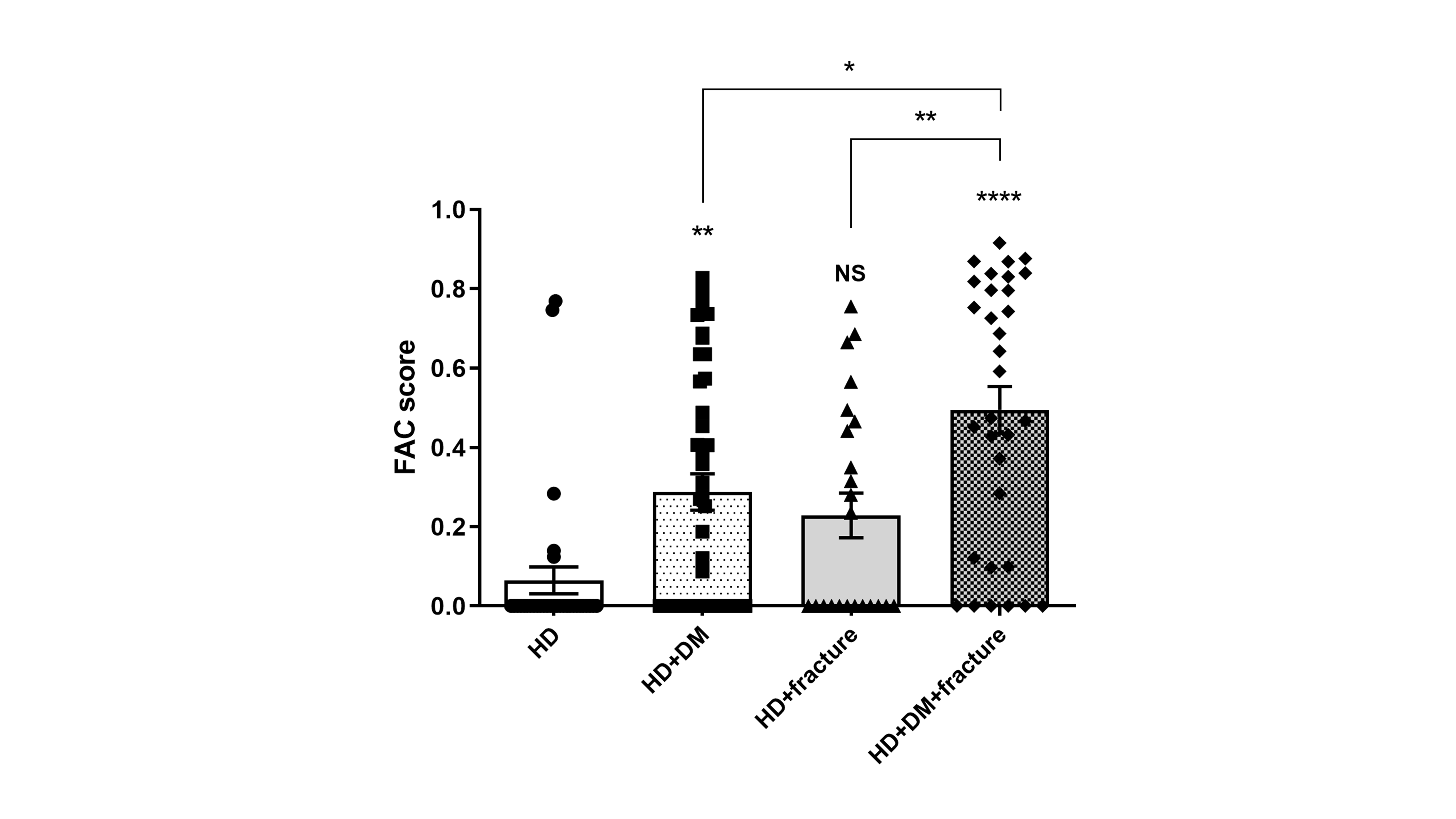

Supplement: Supplementary file 1 — (PNG 903 KB) [file 11657_2025_1536_Fig5_ESM.png]
