# Supplementary material for: Femoral artery calcification predicts hip fracture in maintenance hemodialysis patients
Source: Arch Osteoporos. 2025 Aug 9;20(1):112. doi: 10.1007/s11657-025-01536-1 (PMC12334447; doi:10.1007/s11657-025-01536-1)
Supplement: Supplementary file 3 — (DOCX 15.4 KB) [file 11657_2025_1536_MOESM2_ESM.docx]

**Supplementary information**

**Supplementary Figure 1.** FAC score analysis was subgrouped by the presence or absence of DM or hip fracture. HD patients with DM, especially those with hip fractures, had higher FAC scores than those without DM. Significance was determined by a one-way ANOVA. NS, no significance; * *p* < 0.05; ** *p* < 0.01; **** *p* < 0.0001. Significant difference vs. HD group.

**Supplementary Table 1. Comparison of baseline demographics, clinical features, and laboratory data between HD patients with low and high FAC scores**

| **Variable** | **FAC score** | |  |
| --- | --- | --- | --- |
|  | < 0.27 | ≥ 0.27 | *p-*value |
|  | n = 73 | n = 57 |  |
| **Patient characteristics** |  |  |  |
| Gender |  |  | NS |
| Men, n (%) | 37 (51) | 24 (42) |  |
| Women, n (%) | 36 (49) | 33 (58) |  |
| Age, years | 68.59 ±13.87 | 70.82 ± 10.94 | NS |
| Duration on dialysis, years | 5.14 ± 4.45 | 4.32 ± 4.43 | NS |
| Hypertension, % | 69 | 81 | NS |
| Diabetes, % | 43 | 77 | < 0.0001 |
| Cancer, % | 18 | 7 | NS |
| **Echocardiography** |  |  |  |
| EF, % | 62 ± 10 | 57 ± 13 | NS |
| PAP, mmHg | 39.20 ± 16.84 | 36.67 ± 17.05 | NS |
| **FAC measurement** |  |  |  |
| FAC score (IQR) | 0.00 (0.00 - 0.00) | 0.63 (0.43 - 0.77) | < 0.001 |
| **Laboratory data** |  |  |  |
| Cr (mg/dL) | 7.91 ± 3.69 | 7.31 ± 3.02 | NS |
| Na (mEq/L) | 137.18 ± 3.30 | 136.05 ± 3.45 | NS |
| Ca (mg/dL) | 9.10 ± 0.76 | 9.39 ± 1.16 | < 0.05 |
| P (mg/dL) | 4.93 ± 1.65 | 4.97 ± 1.73 | NS |
| iPTH (pg/ml) | 437.16 ± 414.00 | 347.22 ± 360.76 | NS |
| ALP (U/L) | 85.69 ± 47.98 | 115.66 ± 112.22 | NS |
| AST (U/L) | 23.40 ± 14.94 | 23.40 ± 16.59 | NS |
| Albumin (g/dL) | 3.81 ± 0.56 | 3.60 ± 0.51 | NS |
